# 

**Published:** 1893-04-01

**Authors:** 


### The Hospital

A Weekly Institutional Journal of

### Science, Medicine, Nursing, and Philanthropy.

430

EDITED BY

HENRY C. BURDETT,

AUTHOR OF "HOSPITALS AND ASYLUMS OF THE WORLD: THEIR ORIGIN, HISTORY, CONSTRUCTION, ADMINISTRATION, MANAGEMENT AND LEGISLATION"; "HOSPITALS AND THE STATE"; "PAY HOSPITALS OF THE WORLD"; "COTTAGE HOSPITALS, GENERAL, FEVER, AND CONVALESCENT, WITH FIFTY BEDS AND UNDER": "BURDETT'S HOSPITAL ANNUAL AND YEARBOOK OF PHILANTHROPY"; AND "HELPS TO HEALTH."

ACTING EDITORS:

GEORGE W. POTTER, M.D.Edin.,

AUTHOR OF "MINISTERING WOMEN"; AND NUMEROUS PAPERS ON SCIENTIFIC AND MEDICAL SUBJECTS

AND

P. WATSON WILLIAMS, M.D.Lond.,

PHYSICIAN TO THE OUT-PATIENTS AND FOR DISEASES OF THE THROAT, BRISTOL ROYAL INFIRMARY, ETC.

SURGEON GEN'L'S OFFICE 150278 LIBRARY

Red

INDEX TO VOL. XIV.

(1st April, 1893, to 30th September, 1893.)

LONDON:

THE SCIENTIFIC PRESS (Limited), 428, STRAND, W.C.

1893.

W1
HO69X

### INDEX TO VOL. XIV. 1893.

Aberdeen Royal Infirmary Extension, 320
Aberdeen Hospital for Children, 210
Addenbrooke's Hospital, Cambridge, 280
"A Common Fault," 167
A Co-operative Tour to Rome, 114
Acromegale, 202
A Doctor not a Door-mat, 279
A Lesson for Mr. Stead, 39
A New Order of General Practitioners, 321
A Paternal Kingdom, 145
A Physiological Lantern :—
I. Light, More Light, 277
II. The Baby, its Manners and Morals, 373
Ancoat's Hospital, Manchester, 402
A Reminder, 167
Arctic Explorers, Food for, 178
Around the Hospitals, 2, 18, 34, 50, 66, 82, 98, 146, 162, 210, 242, 258, 274, 290, 306, 322, 338, 354, 370, 402, 418
A Study in Modern Socialism—The Social Horizon, 13
A Suggestion for M.P.'s, 119
Attitude of Great Writers towards Disease :—
XXI. Pepys on the Great Plague, 339
Auchentibber, the Laird of, 375
A Unique City Company, 231
Australia, Medical Practice in, 103
A Year's Work in the Hospitals and Medical Charities of London, 155
Bank Notes, Dangers of, 68
Baron Na haniel Rothschild's Gift, 72
Bath Eye Infirmary, 186, 201
Bathing, Dangers of, 4
Belle Vue Hospital, New York, 253
Bewdley Convalescent Home, 128
Birmingham General Hospital, 2, 30
Birmingham Hospital Saturday Fund, 424
Birmingham New Small-pox Hospital, 352
Blackburn and East Lancashire Infirmary, 66
Blackpool, New Hospital at, 344
Bradford Infirmary, 66
Brighton Life Table, The, 199
Bright's Disease, Milk in, 103
Bristol Eye Hospital, 64, 283
Bristol General Hospital, 26, 82, 169
Bristol Royal Infirmary, 315, 378
British and Foreign Sailors' Society, 120
British Home for Incurables, 280
British Medical Association, 296
British Association, The, 401
Brompton Hospital for Consumption, 59, 328
Cardiff Infirmary, 112
Cancer, Is it Increasing? 135
Cancer Hospital, New Chapel, 216
Can Doctor's Joke? 247
Carbolic Acid, Simple Uses of 217, 234
Central London Throat Hospital, 74
Central London Ophthalmic Hospital, 280
Cerebration, Curiosities of, 423
Charing Cross Hospital, 306
Charnwood Forest Convalescent Home, 392
Chelsea Hospital for Women, 146
Chicago Medical College, 312
Children, Hip Disease in, 215
Cholera, Inoculation, 7
Cholera and the Weather, 372
Cholera Precautions in Villages, 295
Cholera, Tea and Trade, 263
Chronic Hydrocephalus, 347
Church of England Sanitary Association, 376
City of London Hospital, 18
City of London Truss Society, 143, 168
City of London Chest Hospital, 250, 313
Claybury Asylum, 320
Construction Notes, 16, 30, 64, 96, 112, 128, 143, 160, 223, 239, 256, 272, 288, 320, 336, 351, 416, 328
Consumption, Vegetarianism in, 55
Contemporary Theory and Research, 4, 20, 36, 52, 68, 86, 100, 116, 132, 164, 180, 196, 212, 228, 260, 276, 310, 340, 372, 404.
Cookridge Hospital, 256
Corbett Hospital, Stourbridge, 296, 432
Cotswold Convalescent Home, 312
Cottage Hospital, Thames Ditton, 239
Cottage Hospitals, Three, 109
Cromer Convalescent Home, 272
Croydon General Hospital Extension, 223
Curious Survival at Tunbridge Wells, 23
Dartford Cottage Hospital, 416
Death Certification and Still Born Babies, 119
Dental Schools in London, 400
Diet in Disease (continued from Vol. XIII :)
XVIII. Indigestion, 5
XIX. Indigestion (*continued*), 21
XX. Under-feeding and Over-eating, 53
XXI. Under-feeding and Over-eating (*continued*), 117
XXII. Under-feeding and Over-eating (*continued*), 165
XXIII. Food for the Aged, 181
XXIV. Uric Acid as a Cause of Disease and its Prevention by Diet, 197
XXV. On Thinning and Fattening, 213
XXVI. On Thinning and Fattening, (*con.*) 261
XXVII. Ulcer of the Stomach, 291
XXVIII. Chronic Bright's Disease, 323
XXIX. Chronic Bright's Disease (*con.*) 355
Devonshire Hospital and Buxton Bath Charity, 274
Doctors and Death, 39
Doctor's Defences, Mr. Jonathan Hutchinson, on, 193
Doctors, the Ups and Downs of, 263
Do we Starve Our Children? 311
"Drink Crave Cure," The, 231
Drink Question in Switzerland, The, 85
Dr. Barnardo's Homes, 184
Dunfermline Cottage Hospital, 351
Earlswood Asylum, 88
East London Hospital for Children, 77
Editor's Letter Box :—
Uses of Hospitals, 172; A Reminder for Absentees, 191; The Disinfection of Wounds, 191; The Opium Question, 191; The Treatment of Disinfection of Scarlet Fever by Antiseptic Inunction, 208, 224, 236, 256; Sanitas Oil and Inunction, 288, 303; Duodenal Indigestion, 316, 332; Practical Experience in Poultice-making, 368; Fish and Cholera, 399; How to Diminish the Number of Doctors, 428
Electrical Treatment, Arrangement for, 233
English and American Doctors, 241
Equal Service, Equal Honour, 65
Experiment and Experience, 1
Falmouth, New Hospital for, 112
Famous Poisoners in Fiction :—
I. Introductory, 293
II. A Daughter of Darkness, 307
III. Catherine, 341
IV. A Royal Poisoner, 358
V. The Poison Maid, 405
VI. A Mother's Love, 421
Feeble-minded Girls, Laundry Work for, 327
Feeble-minded, The Care of :—
I. In Childhood, 366
II. In Later Life, 431
Female Sanitary Inspectors, 228
Festival Dinners and Meetings, 64, 80, 95, 111, 127, 144, 160, 172, 206, 222, 239, 255, 271, 287
Fighting the Microbe, 308
Fletcher Convalescent Home, Cromer, 72
Foresters' Convalescent Home, Clent, 432
Foods for Arctic Explorers, 178
Fractures of Nose, 218
From Chemist's Shop to Physic's Throne, 387
George Smith, The Man or His Monument, 135
Glasgow Western Infirmary, 233
Gleanings in Science and Medicine, 6, 22, 38, 54, 70, 84, 102, 118, 134, 166, 182, 198, 214, 230, 244, 278, 292, 324, 342, 356, 374, 406, 422
Gordon Boys' Home, 280
Gravesend Hospital, 226
Great Achievements under Suffering—
I. Professor H. Fawcett, 69
II. The Blind Poet, 309
III. The Deaf Musician, 371
IV. The Blind Observer, 419
Guy's Hospital, new buildings opened, 216
Guy's Hospital, 269
Habitual Drunkards, 180
Halifax Infirmary, 200, 232
Health Resorts, A Protest, 295
Heart Disease, Cycling and, 263
Herbalism in Yorkshire, 55
Herbal Simples, History and Capabilities of—
Garlic and Onion, 262, 326; Gooseberry, 420
Hip Disease in Children, 215
Holbeck Nurses' Home, 96
Homes for London Working Boys, 232
Home or Workhouse? 343
Hospital Administration (*continued*) :—
Hospital Accounts, 45; Cost of Royal Portsmouth Hospital, 61; General Hospitals, 333; Special Hospitals, 349; Children's, 381; Lying-in and Women's, 413; Ophthalmic and Miscellaneous, 429
Hospitals Associations, The Work of the, 305
Hospital Clinic—
Treatment of Abscesses, 106
Treatment of Acute Rheumatism, 27
Treatment and Disinfection of Scarlet Fever, 299, 412
Treatment and Diagnosis of Cystic Tumours in Scalp, 330
Treatment of Ague, 411
Treatment of Appendicitis, 297
Treatment of Asthma, 59
Treatment of Bronchitis, 346
Treatment of Cancer of Breast, 266
Treatment of Caries of Spine, 425
Treatment of Chronic Ulcer, 361
Treatment of Cleft Palate, 345
Treatment of Club Foot, 41, 169
Treatment of Cold Abscess, 42
Treatment of Compound Fractures, 329
Treatment of Cosmetic Defects, 186, 201
Treatment of Cough as Symptom in Phthisis, 265
Treatment of Diabetes Mellitus, 331
Treatment of Flat Foot, Bow Legs, and Knock Knee, 25
Treatment of Fracture and Dislocation of Spine, 409
Treatment of Fractures of Nose, 218
Treatment of Fractured Patella, 26
Hospital Clinic (*continued*)—
Treatment of Gall Stones, Surgical, 105
Treatment of Glaucoma, 283
Treatment of Hammer Toe, 377
Treatment of Indigestion, 268
Treatment of Infantile Paralysis, 411
Treatment of Ingrowing Toe-nail. I.—Pathology, 426
Treatment of Injuries to Scalp, 90
Treatment of Intestinal Cancer, 187
Treatment of Lateral Curvature of Spine, 281
Treatment of Otorrhœ (*continued*), 185
Treatment of Pernicious Anæmia, 249
Treatment of Pleural Effusion, 11
Treatment of Pleurisy and Empyema, 282, 298
Treatment of Pulmonary Phthisis, 235, 250, 313
Treatment of Rheumatism, 57
Treatment of Rhinitus, 74
Treatment of Scirrhus of Breast, 12 1
Treatment of Senile Gangrene, 91
Treatment of Simple Fracture of Femur, 9, 73
Treatment of Simple Fracture of Tibia and Tibula, 137
Treatment of Stiff Joints, 267
Treatment of Stone in Bladder, 315
Treatment of Tonsillitis, 378
Treatment of Tubercular Enteritis, 361, 378
Treatment of Tubercular Joints, 89
Hospital Construction, 29, 94, 123, 285, 317, 365
Hospitals in India, 350
Hospital Meetings, 318, 382
Hospitals of New York, 205
Hospitals of the World, IV. Nursing Systems Abroad, 47
Hospital Opinion, 80
Hospital Saturday Fund, 46, 328, 344, 424
Hospital Sunday, 189, 168
Hospital Sweetness and Light, 147
Hospital for Diseases of Throat, 18
Hospital for Sick Children, Great Ormonde Street, 88, 290
Hospital Ship at Ipswich, 416
Hostel of St. Luke, 40
How a Lady may become a Doctor, 389
How to "Dish" the Food Adulterator, 391
How to Make Both Ends Meet, 150
How to Keep Well in Africa
I. Prevention, 101
II. Cure, 133
Hudder field Infirmary, 91, 160, 306, 409
Hunter Memorial at St. George's, 216
Hutchinson, Mr. Jonathan on—
Doctors' Defences, 193
Huxley's Logic and Life, 129
"I Decline to Meet You," 215
Iced Water, 340
Illness, The Advantage and Hygiene of, 403
Ilkeston, New Hospital at, 112
Illinois Training School for Nurses, 237
Ingham Infirmary, South Shields, 402
Infantile Paralysis, 411
Infants and Mail Carts, 247
Influenza, the Mystery of, 353
In-growing Toe Nail, 426
Is Massage a Swindle? 7
Insane, The Story of from Year to Year, 110, 142, 270, 319, 334, 366, 414, 429
Insanity, The Beginnings of, 23
Innunction, Methods of Using, 299
Isolation Hospital, Norwich, 30
Jewish Convalescent Home, Hove, 96
King's College Hospital, 264
Klein on Cholera Inoculation, 7
Lady Brassey's Home, 120
Lancaster New Infirmary, 223
Larbert Fever Hospital, 416
Larbert Hospital for Insane, 312
Laziness as a Cure, 71
"Leeds Hospital Magazine," 408
Leeds General Infirmary, 73, 105
Leeds and her Back-to-Back Houses, 87
Leicester M. C. and Fever Hospital, 216
Leprosy Commission, A, 55
Leprosy in Trinidad, 359
Lewes Dispensary, 370
Liberator Relief Fund, 216
Liverpool Royal Infirmary, 11
London Editors and London Hospitals, 289
London Fever Hospital, 274
London Homœopathic Hospital, 184, 338
London Lock Hospital, 162
London Temperance Hospital, 34
London Young Women's Christian Association, 392
London Hospital, 104, 106, 170, 266, 312, 329, 361
London Hospital, The Death at, 415
London Hospital and the *Pall Mall Gazette*, 301
London Poor and Their Medical Needs, 31
London's Quinquennial Appeal, 119
Luxor Hospital Egypt, 152
Manchester Southern and Maternity Hospital, 232
Mary Wardell Home, 104
May the Children Help? 149
Meath Home for Epileptics, 248, 264
Meath, Lord, Physical Educator, 423
Medical Velocipede Club, 168
Medical Education, The Cost of, 388

Medical Protection and Medical Defence, 183
Medical History from the Earliest Times (*continued*)—
XLVIII. Paracelsus, 3
XLIX. Similars, Sympathy, and Signatures, 19
L. The Great Anatomists, 35
LI. The Reform of Surgery, 51
LII. Medical Practice in the Sixteenth Century, 67
LIII. Harvey and the New Physiology, 83
LIV. Van Helmont. 99
LV. The Chemical School of Medicine, 115
LVI. The Iatromechanical School, 131
LVII. The Cl nicians, 163
LVIII. The Clinicians (*continued*), 179
LIX. The Animists, 195
LX. The Vitalists, 211
LXI. The Organicists, 227
LXII. The Surgery of the Eighteenth Century, 243
LXIII. The Brunonian System, 259
LXIV. The Origin of Modern Medicine, 275
Mental Chronometry, 116
Metropolitan Asylums Board, 72
Metropolitan Drinking Fountains Association, 168
Metropolitan Hospital, 2, 34
Metropolitan Hospital Sunday Fund, 300, 304
Metropolitan Medical Schools, 385
Miller Hospital and Royal Kent Dispensary, 402
Mirfield Memorial Hospital, 30
Modern Social sm, A Study in, 13
Monkey Language, 196
Morley House Convalescent Home, St. Margaret's Bay, 376
Moral and Physical Analogy, 343
More Wicked than Sacrile e, 407
Mothers' Seaside Convalescent Homes, 168
Mr. Alabone and his Editorial Friends, 199
Mr. Balfour and "Research," 70
Mr. Stead, A Lesson for, 39
"My Lord Porridge," 375
Mulattoes, The Offspring of, 20
National Home Reading Union, 216
National Hospital for the Paralyzed and Epileptic, 82
Naphthols, as Intestinal ante-infectants, 192
Nebuchadnezzar's Feast, 37
Nerves, New Enemie of, 36
Neolithic Surgery, 215
New Homœopathic Hospital, 200
New Hospital and Dispensary, Scarborough, 352
New Hospital for Women, 146
New Police Convalescent Home, Hove, 280
New Sisters' Hospital, St. Albans, 240, 366
New York Ladies' Health Protection Society, 152
New Appliances, Drugs, and Things Medical, 12, 28, 44, 95, 123, 124, 139, 140, 158, 203, 204, 220, 251, 252, 268, 284, 316, 332, 363, 364, 379, 399, 427, 428
Newca tle C ty Home for Incurables, 344
Newcastle Eye Infirmary, 220
Newcastle-on-Tyne Throat and Ear Hospital, 185
New Treatments, New Dangers, 183
Norfolk and Norwich Hospital, 162
North Devon Infirmary, 370
North London Con umption Hospital, 18, 306
North-Eastern Hospital for Children, 93
Nose, Fra ures of, 218
Notes and News, 8, 24, 40, 56, 72, 88, 104, 120, 136, 152, 168, 184, 200, 216, 232, 248, 264, 280, 296, 312, 328, 344, 360, 376, 392, 408, 424
Nuneaton Cottage Hospital, 424
Nurses' Home, Derbyshire Infirmary, 416
Nurses, a Terrible Warning to, 199
Oldham Infirmary, 98, 128
Our Weaker Brethren, 229
Overcrowding in Lanarkshire, 327
Owens College Hospital, Manchester, 24
Oxford, a Childish Quarrel at, 183
Paddington Green Children's Hospital, 282, 298, 322
Pains, Brains, Gains, 151
Pan-American Medical Congress, 312
Paris Municipal School of Physics and Chemistry, 120
Parents and School Boys' Loyalty, 359
Parke, The Late Surgeon-Major, 386
Parkwood Convalescent Home, Swanley, 336
Pawnbroker, The Apothesis of the, 325
Pental as an Anæsthetic, 310
Physio ogical Pessimism, 103
Poisoned Arrows, 180
Poor Pay, Bad Work, 247
Poplar Hospital for Accidents, 50
Poplar Hospital, 216
Porthcawl Convalescent Home, 376
Practical Departments:—
Smoke Consuming Stoves, 30; Fire Escapes, 46; I. Invalid Chairs, 63, II. Invalid Chairs, 79; L. K. Batteries, 109; Bed Table, 126; Reading Stands, &c., 142; The "Bra oo" Window, 159; Warren's Cooking Pot, 190; The "Gourmet" Boiler, 190; Ward's Ambulance Trolly, 207; Hospital Furniture, 207; Furniture for Nurses' Rooms, 221, 271; Hygienic Tea Pot, 238; Toilet Tray, 238; Ward Waggons, 254; Chamber Portable Closet, 254; Ambulance Equipments, 335; Gas Stoves, &c., 351; Emergency Case, 351; Sponges, 367; Boot Warmer and Drier, 415; Dressing Waggon, 431
Practical Education, 327
Practitioners Bookshelf, The 12, 43, 76
Prescribing in the Dark, 17
Presidency of R.C.P, 7
Proposed Central Hospitals Board for London, 80
Prolonged Incubation of Mumps, 68
Public Health, King's College and, 423
Public Health, Relation of Hospitals to, 245
Quacks, How to Detect, 375
Queen's Hospital, Birmingham, 18, 210
Radcliffe Infirmary, Oxford, 79, 200
Rags and Reason, 23
Reckless Cycling, 391
Recreative Evening Schools Association, 360
Recreation for Middle Age, 81
"Research," Mr. Balfour and, 71
Rhinoscopy, 107, 122, 138, 169
Rich Deserters at West End Churches, 295
Ridiculous Fellows of the Royal Society, 167
Royal Berkshire Hospital, 162
Royal Free Hospital, 210
Royal Free Hospital and Cubitt's Fire, 216
Royal Hants County Hospital, 338
Royal Hospital for Incurables, Putney, 88
Royal Infirmary, Newcastle-on-Tyne, 50
Royal National Hospital for Consumption, 152
Royal Portsmouth Hospital, 290
Royal South London Ophthalmic Hospital, 136
Royal United Hospital, Bath, 240
Royal Victoria Hospital, Montreal, 184, 285
Royal Westminster Ophthalmic Hospital, 136
Royal Frederick Hospital, Copenhagen, 187
Royal Hospital for Diseases of Chest, 2, 235, 265, 346
Royal Infirmary, Edinburgh, 42, 90, 121, 242, 249, 328, 330, 345
Royal Orthopædic Hospital, 25, 41, 267, 281, 377, 425
Royal Southern Hospital, Liverpool, 57, 89, 411
Rumworth Hospital, 64
Rural Sanitary Authorities' Emergency Hospital, Rumworth, 64
St. George's Hospital, 98, 143
St. John's Ambulance Brigade, 296, 312
St. Mary's Hospital, Manchester, 96
St. Mary's Hospital, 27, 34, 200
St. Thomas's Hospital, 424
Sanitary Institute, Parkes Museum, 424
Scarlet Fever, Innunction in, 412
Scents as Antiseptics, 260
School Hygiene, 33
Scraps and Gleanings, 16, 32, 48, 64, 80, 96, 112, 128, 144, 160, 192, 208, 224, 240, 256, 272, 288, 320, 336, 352, 368, 416, 432
Seaman's Hospital, The, 242
Sea Shells and Children's Scrap Book Mission, 344
Shaw Convalescent Home, Bearsden, Glasgow, 143
Should Adults be Revaccinated? 49
Simultaneous Movements, 404
Sick Room, The, 311
Skin Grafting, Methods of, 170
Skegness Convalescent Home, 200
Smithsonian Institute of America, 248
Snoring, A "Cure" for, 87
Some Neglected Medical Obligations, 113
Stands Edinburgh for Untrained Nurses? 39
Stertorous Breathing, Remedy for, 52
Stornoway Hospital, 224
Summer Cobwebs and the Broom, 231
Sunderland Infirmary, 322
Sussex County Hospital, 9, 137
Tea Drinkers, A Nation of, 359
Temperature, Food and Dress, 337
The Afternoon Nap, 71
The Danger of "Safe" Hypnotics, 311
The International Hospital Congress at Chicago, 161
The London and Counties Medical Protection Society (Limited), 104, 348
The Morell Mackenzie Controversy, 177
The New Coachman of the British Association, 407
The *Pall Mall Gazette* and the London Hospital, 273
The "Reckoning," 87
The Royal British Nurses' Association, 194.
The Royal Wedding, 209
The Student at the Apex, 417
Thin Skins, 279
Tobacco as a Microbicide, 100
Transplantation of Frog's Skin, 86
Unhealthy Employments, 164
University College Hospital, 34
Vaccinal Immunity, 132
Vaccination, 119
Vaccination, A Striking Experiment in, 279
Victoria Hospital for Children, Chelsea, 392
Vivisection, Is It Moral? 407
Volunteer Medical Staff Corps, 264
Wanted, a Medical Defoe, 391
Wallsend Fever Hospital, 288
"Washed Daily on the Allotment System," 135
Warrington Infirmary, 240
Wasps' Nurseries, 341
Watford Cottage Hospital, 136
What becomes of Stillborn Children, 294
What he Hospitals do for the Public, 131
Where are the Bri ish? 343
Why are Parisians Undersized? 4
Why do Doctors not Advertise? 97
Will Specialists become Extinct? 257
Willesden Cottage Hospital, 272
Within the Hospitals:—
The East London Hospital for Children, 77; The North-Eastern Hospital for Children, 93; Three Cottage Hospitals, 109; The Hospitals of New York, 205; Newcastle Eye Infirmary, 221; The Illinois Training School for Nurses, 237; Belle Vue Hospital, New York, 253; Guy's Hospital, 269
Women's Work in South Africa, 357
Workers and Weariness, 369
Worcester General Infirmary, 50
York County Hospital, 64

### THE SPECIAL NURSING SUPPLEMENT (MIRROR).

A Capital Suggestion, clii
A German Hospital, xliv, liv, lxv
Alumnæ Associations, Benefits of, ccxxiv
Ambulance Service in Italy, cxi
America, Training Schools in, cxxi, cciii
An American Novelty, clxxxi
An Appropriate Suggestion, cxxi
A Neglected Field, lxxxvii
An Important Decision, ccxli
An Infallible Cough Mixture, viii
A Novel Competition for Nurses, clxxvii
An Underpaid Amateur, clxx
A Plea for Sick Paupers, i
A Royal Guest, i
A Royal Inspector, xi
A Royal Visit, li
A Sad Tragedy, cxxvii
Associations for Supply of Cottage Nurses, cxciv
Associations for Nurses for the Insane, cvii
At Home and Abroad, ccxli
At Less than Cost Price, ccxli
Attainable Perfection, vi, xxv, xxxv
Attributes to Aim a , xi
A Useful Association, ccxiii
A Useful Suggestion, cxxxvi
Australia, ccli
Australia, Nursing in, xxvii
Australia or New Zealand? clxxvi, covi
Australia, Prince Alfred Hospital, Sydney, xxiv
Australian Notes, ccxxviii, ccxxxvii
Bangor, Progress at, clxxxi
Berlin, Trained Nursing in, ccliii, ccliii
Bexhill, Home at, ci
Book World for Women and Nurses, ix, xix, xxix, xxxix, xlix, lix, lxix, lxxix, lxxxix, xcix, cix, cxix, cxxix, cxxxix, cxlix, clix, clxix, clxxix, clxxxix, cxcviii, ccix, ccxix, ccxxix, ccxxxix, cclix, cclxix
Borrowed without Acknowledgment, vii
Braintree, District Nursing at, cxxv
Bristol Royal Infirmary, ccxxi
British Columbia, clxviii
Busy Bees at Barrow, cxi
Cambridge, xxi
Cape Mission, ccvi
Ceylon, Civil Hospital, Colombo, cv
Charity and Cheerfulness, xxi
Chicago, ccxxi, cxci
Chicago, Nursing Subjects at, xlvi
Children's Hospitals, Training in, xxxvi
Cholera, Notes on, ccxiii
Cholera, Nursing in, lxvii
Cholera, Precautions Against, cxxxvii
Church of England Sanitary Association, clxvi
Clapham Maternity Hospital, ccxi
Clothing for Infectious Hospitals, ccxi
Colombo, The Leper Asylum at, lxxxiv.
Conscientious Disinfection, lxxiv
Consumption, Infectiousness of, xlviii
Consumption, Management of
VIII. Occupation for the Consumption, iii
IX. The Feeding of Consumptives, xiii
Control of Nursing, The, cci
Convalescence, How to Secure, ci
Convalescence, Provision for, cxli
Convalescent Home, vii
Cooking, Scientific, cclxi
Co-operation, xvii
Cork Hospital for Women and Children, cclv
Cornish Holiday Resorts, ccxv
Cornwall, ci
Cornwall, A Holiday in, clxxvi
Cottage Nursing, clv, cxci, ccxxxv
Dangerous Playground, lxi
Define Duties, lxi
Disabled Nurse, The, i, xxi, xxxi, xli, lxi, ci, cx i, clii, clxxxi
Discourtesy to a Matron, cxxi
Disease, Curiosities in, clxx
District Nursing, lv, clxxxiv, ccxlvi, cclxvi
District Nursing at Melbourne, li
District Nursing at Stalybridge, clii
District Nursing at Yarmouth, lxxxi
District Nurses in America, clxi
Doctor Hadden, The Late, xci, ci
Does Massage Pay? lxxxi
Dublin, xli

Dublin, Good Nursing in, xxxiv
Dufferin Hospital, Allahabad, lxxxiv
East London Hospital for Children, xxxvi
Economy at Melbourne, xi
Educational Standards for Nurses, cliv, clxxiv
Elastic Limits, ccli
Emergencies, Education in Dealing with, ci
Emergencies Intelligently Met, clii
Endowed Bed, The, xi, xxi, lxi, lxxi, lxxxi, xci, cxli
English and American Training, cxci
English Nurses in Paris, ccvi, ccxli
Everyday Dangers, cxci
Europeans in India, cxi
Fair and Unfair Expenses, xciv
Faithful Service, i
False Economy, lxxxi
Fever Nursing, li, lxxxv
Fleeting Glories, xci
Free Conveyance, Bereavement, and Bitterness, i
Free Lectures, clxxxvii
Free Libraries, i
Freedom and Fresh Air, xci
French Doctor's Waiting Room, A, ccli
Gaol, A Convalescent in, lxi
Germany, The Work of Deaconesses in, ccxxxiv
Gifts Easy to Choose, ccxli
"Giving In," On, ccxiv
Good Management at Perth, xxxi
Good News for Kandy, xi
Gratitude, i
Groat's Worth of Wit, A, cxxviii
Her Better Self, xxxviii, xlviii, lviii
Hero from a Convalescent Home, A, ccxxi
Hints for Nurses, xvi, xxv, lxvi
Hints from America, xvii
Holy Week, Thoughts About Pictures for, vi
Hospitality to Nurses, cclxi
Hospital Discipline, cxxvii
Hospital Experiences in Umtali, Manicaland, xxxiv, lxxxvi
Hospital for Soldiers' Wives, clxxvi
Hospital for the Indians, British Columbia, clxviii
HOSPITAL, THE, Endowed Bed, xi, xxi, lxi, lxxi, lxxxi, xci, cxli
How Not to Nourish Babies, xxxv
How to Spread Infection, xi
Illinois Training School, cci
Incompetent to Criticise, clxxxi
Incurables, A Home for, ccxxvi
India, xvii, ccli
India, Drawback to a Hill Station, cxxxvi
India, Mahableshwar, cxxxvi
India, New Nursing Arrangements in, cxxxi
India, Nursing in, xli, lxxxiv, xciii, xciv, civ, cxiv, clxxxi
Indian Nursing Service, The, ccxi
India, Upcountry Nursing Association, xlvii
Infectiousness of Consumption, xlviii
Ingenious Disinfection, i
"In Hospital," cxxi
Insane, Association of Nurses for the, cvii
Insanity Among Coloured Races, cclxiii
In Service, but not of Service, clxx
Intelligent Precaution, ccxli
Interesting Cases, cxi
Invalid Children's Aid Association, The, xxxi, lxi
Japan, cvi
Jottings from the Punjab, xiv
Jubilee Institute at Taunton, The, xxxvi, xlv
Kilmanock, xxxi
Ladies in the Nursery, ccxxxi
Ladies' Sanitary Association, lxxxi
Lady Nurses for Officers in India, clxxxv
Lady Roberts' Nursing Fund, cxxxvi
Leeds, Workhouse Nursing at, ccxi
Leper Asylum at Colombo, lxxxiv
Lepers in New South Wales, lxi
Liberality at Burton-on-Trent, i
Lifting of Patients, The, xxxv, lvi
London Hospital, clxxxvi, cxcvi
London Skin Hospital, cxv
Lord Winchelsea's Scheme, ccxxxi
Madras, A Nursing Home, ccxxxii
Male Nurses, clxi
Male and Female Nurses in German Catholic Orders, cxcv
Management of Consumption, Explanation, xvii
Manchester Infirmary, cvi
Manx District Nursing, xxxi
Manicaland, Hospital Experiences in, xxxiv
Maternal Sanitation, xlviii
Maternity Nurses, ccxxi
Measles, lxxv, lxxxv
Medals at Gravesend, clii
Medical Missions, clii
Medico-Psychological Association, cxvi
Mental Nurses, Instruction for, ci
Merits and Demerits, clxxxi
Methodist Episcopal Hospital, Philadelphia, cclxvii
Metropolitan and National Nursing Association, lxxv
Middlesex Hospital, lxxv
Midwives, Registration of, cxcvi, cciv
Midwives, Select Committee on, clv
Military Tournament Hospital, cv
Miners at Windor, lxi
Ministering Children at Lancaster, xci
Misapplied Economy, xli
Miss Nightingale's Paper, clxi
Miss Victoria Jones. ccxxi
Mistaken Economy, cclxii
Moderate and Immoderate Exercise, xli
Modern Nurses, cclxiv
More Girl Nurses, cxxxvi
More Work for Competent Women, civ
Mrs. Garrett-Anderson, Presentation to, cxv
Much too Young, ccxli
Muses' Looking Glass, The, xviii, lxviii, lxxviii, lxxxviii. cxviii, cxxxviii, clxxviii, clxxxviii, ccviii, ccxviii, ccxxviii, ccxxxviii, ccxlviii, cclviii, cclxviii
Neglected Paupers, cxxi
New Day Nursery, Plaistow, lvi
New Jersey, U.S.A. Nurses in, xxxvii
News, Nurses and Clubs, xxvii
New South Wales, Lepers in, lxi
New York, clxxxi
New York Training School, clxxxvii
Nightingale Fund, The, ccxxi
Noble Example, A, clxi
Notes and Queries, v, xv, xxv, xxxvi, xliv, lviii, lxvi, lxxvii, lxxxvii, xcvii, cvii, cxvii, cxxviii, cxlviii, clvii, clxviii, clxxviii, clxxxviii, cxcvii, ccvii, ccxvii, ccxxvii, ccxxxviii, ccxlvii, cclviii, cclxviii.
Novel Training Schools, xi
Novelties for Nurses, iii
Nurse Dolls, xli, li, lxi, lxxxi, xci, ci, cxi, cxxi, cxxxv.
Nurses and Cholera, ccxviii.
Nurses and Fire Drill, cxci,
Nurses as Missionaries, ccxi.
Nurses as Patients, cvi
Nurses as Scrubbers, xli
Nurses at Berlin, xi
Nurses for Norfolk, vii
Nurses' Home, Competition for, cci, ccxxi
Nurses' Homes, cc, ccxiv
Nurses, Increased Accommodation for, clxxxi
Nurses in Wales, xi
Nurse Snorers and Others, lvi
Nurses, Pensions for, lxiv
Nurses, Quarantine for, lvii
Nursing at Dundee, ccxxi
Nursing at Hinckley, lxxxi
Nursing at Lynn, ccxi
Nursing at Natal, iv, xiv
Nursing at Southampton, lxxvii
Nursing at Winchester, cci
Nursing in a Cottage Hospital, ccxlv
Nursing in Australia, xxvii
Nursing in India, xciv, civ, cxiv
Nursing in Paris, cclv, cclxiv
Nursing in Plaistow, xxi
Nursing in South Africa, ccxlv, cclxv
Nursing Sisters, ccxi
Nursing Subjects at Chicago, xlvi
Nursing Uniforms, ccvi, ccxxvi, ccxxxvi, cclvi
Obvious Dangers, cclxi
Old Age, Provision for, li
On Land and Sea, c xi
Opening, A New, cci
Orange, U.S.A., Nurses' Home for, xxi
Ordinary Nurses, i
Our American Letter, clxxv
Our American Sisters, ccli
Our Examinations, xxvi, lxxvi, cxxvi, clxxxv, ccli
Our Needlework Competition, ccxli
*Pall Mall Gazette* and London Hospital, clxiv
Parish Nurse, The, ccxv, ccliv, ccxxiii
Parisian Precocity, lxi
Paying Cottage Hospital, A, cvi
Pennies well Applied, xci
Pension and Sick Assurance, cclxi
Pensions for Nurses, lxiv
People who "Want to Know," ccxli
Permanent Home, A, cxxxi
Philadelphia, Notes from, ccxxxvii, cclxvii
Pity the Children, lxxi
Plague of Flies, lxxxi
Plain and Unattractive Duties, cxxxi, cxlvii
Poor Economy, clxx
Poplar Hospital for Accidents, xxxi
Position of Nurses, The, ccxiiv
Practical Information, ccxxiii
Practical Sanitation, xxi
Practising and Preaching, cxli
Press Notices, clxxvi
Prince Alfred's Hospital, Sydney, xxiv, xcv.
Privy Council and the Charter, The, xxi
Probationers at Bristol, cxi
Probationers, Examination for, xcvii
Proposed Nurse Training in Ireland, clxx
Prosperous Irish Hospital, A, xxxi
Provident and Improvident, cxli
Qualified Medical Women, cxli
Quarantine for Nurses, lvii
Queen's Nurses, clxxxv
Queen's Nurses at Darlington, clii
Queen's Nurses at Portsmouth, li
Queen's Nurses at Ryde, xli
Queen's Nurses, Rest for, cxi
Queen Victoria Jubilee Institute, lxvii, cxiv, cxlvii
Rabies and Glanders, clvi
Reading to the Sick, vii, xvii, xxv, xxxvii, xlvii, lvii. lxxvii, lxxxvii, xcvi, cvii, cxvii, ccxxvii, cxxxvii, clvii, clxviii, clxxvii, clxxxvii, cxcvii, ccxvii, ccxxvii, ccxxxvii, ccxlvii, cclxvii.
Red Cross Flower Mission, The, clii
Registered Nurses, ci
Registration of Nurses, xcv
Rescued, xxviii
Rest and Recreation, xxi
Rio Janeiro, lxxxi
Robben Island, The Lepers, cxlvii
Royal British Nurses' Association, clxxvi, xxxvii, cxxiii, cxxvi
Royal National Pension Fund, xxi, cvi, cxci
Royal Patronesses, i
Royal Visit, A, clxi
Royal Visit to Clerkenwell, cxxi
Royal Wedding, The, xci, ci, cxi, cxxi, cxxxi
Rural Nursing in Lincolnshire, clxxxii
St. Luke's House, cci
St. Mary's, Plaistow, lvi
Sanitary Law, lxxv
Sanitation for Nurses:—
I. Infectious Diseases, xxiii
II. Notification in Foreign Countries, xxxiii
III. Notification in Foreign Countries, xliii
IV. Sweden, liii
V. Italy, lxiii
VI. Spain, lxxiii
VII. Finland, lxxxiii
VIII. Vaccination in various Counties, xciii
IX. Some Recent Sanitary Provisions, ciii
X. Some Recent Sanitary Provision, cxiii
XI. Sanitary Provisions in Foreign Countries, cxxxiii
XII. Disinfection and Disinfectants, cxliii
XIII. Disinfection and Disinfectants, cliii
XIV. Heat as a Disinfectant, clxiii
XV. Apparatus for Disinfection, clxxiii
XVI. Methods of Disinfection in Foreign Countries, clxxxiii
Scientific Cookery, cclxi
Scotch Branches of the Institute, cxxxi
Scotch Workhouse Nurses, cxxxi
Sea Breezes, cxxi
Self Help, li
Scent, xcviii, cviii
Serious Warning, A, lxxi
Short Items, i, xi, xxi, xxxi, xli, li, lxi, lxxi, lxxxi, xci, ci, cxi, cxxi, cxxxi, cxli, clii. clxi, clxx, clxxxi, cxci, cci, ccxi, ccxxi, ccxxxi, ccxli, ccli, cclxi
Should Nurses be a Distinct Caste? cxcvi
Sickness and Sewerage, ccxxxi
Sick Paupers, A Plea for, i, clxi
Sick Room Cookery, cxli
Sick Room Visiting, cxi
Silent Sufferers, cxci
Small-Pox, cxlvii
Somerset Hospital, Cape Town. cxxxiv
South Africa, Nursing in, cxxxiv
Spain, lxxxv, cxxvi
Stirling, xli
Sunderland, Advancement at, cxci
Surgical v. Medical Nursing, cxxv
Tailor and Nurse, cxci
Teachers and Taught, ccli
Tea Drinking, ccxxxiv
Test Questions. xvi
The First Certificate, ccxi
The Florence Nightingale Pledge, lxxi
THE HOSPITAL Competition for Nurses' Homes, cci
The Vaterlandische Frauenverein, xlvi
The Young Nurse, xvii
Thoroughness, cci
Trained Nursing at Berlin, ccxliii, ccliii
Training at St. Marylebone Infirmary, ccxxxi
Training in Children's Hospitals, cxxxvi
Training Schools in America, cciii
Trains and Trained Nurses, lxxxi
True Gentlewomen, ccxxxi
"Truth about London Hospital," ccxxv
Twenty-eight Years After," xli
Uniform v. Registration, clxx
University College Hospital, cxi
Unprofessional Criticisms, cxciii
Use and Abuse of Uniform, cxciii
Valuable Lectures, clxxxi
Valued Workers, i
Value of a Signature, The, cxli
Vancouver, A New Home at, clxxxi
Victoria House, Berlin, The, ccliii
Village Nurses, xli, ccxxxi
Vivisection, Is it Justifiable? cclvi
Wales, cxi
Wants and Workers, viii. xviii, xxv, xxxviii, xlvii, lvi, lxiv, lxxvii, xcvi, cv, cxiii, cxxxviii, clxxvi, clxxxiv, ccxvii, ccxlvi, cclvi, ccliii
Ward Nurses, A Hint to, cclxiii
Warnings, lxvi
Watford District Cottage Hospital, cclxvi
"What's in a Name!" cxxi
Where to Go, xiv, xlvii, lvii, lxvi, lxxxv, cxvii, cxxvi, cxxxvi, cxliv, clxvi, cclxv
Who Supplies the Drugs? ccxxxvi
Wise Local Government, cxxxi
Woman's Share, xi
Women Inspectors, xxv
Workhouse Infirmary Nursing, ccxi
Workhouse Infirmary Nursing Association, cv
Workhouse Sick Wards and Poor Law Infirmaries, ccxxxi
Workhouses, Nurses in, cxvii, clii, ccviii, ccxvi, ccxxv
Work in Scotland, cclxi
World of Nurses, The, ccvi, ccxvi, ccxxv
Worth Consideration, clii
Worthy of Imitation, ccli

Printed by W. H. AND L. COLLINGRIDGE, "City Press," 148 and 149, Aldersgate Street, London, E.C.

**OUR NEW OFFICES.**

428, Strand, W.C., will henceforth be the Office of this Journal.

### NOTICE TO CORRESPONDENTS.

All MS., Letters, Books for Review, and other matters intended for the Editor should be addressed The Editor, The Lodge, Porchester Square, London, W.

The Editor cannot undertake to return rejected M.S., even when accompanied by stamped directed envelope.

**Notice to Advertisers and Subscribers.**

All Advertisements, Orders for Copies of The Hospital, and Business Communications should be addressed to The Publisher, *not to the Editor*, The Hospital, 428, Strand, W.C.

The Cost of Subscription, post free, is as follows:—Per week, 2½d.; per quarter, 2s. 6d.; per half-year, 5s.; per annum, 8s. 6d. Foreign;—Per annum, 10s. 8d.; payable, in all cases, in advance.

**"Burdett's Hospital Annual," 1893.**

Fourth year of publication. 700 pp. Boards, gilt lettering. A most complete guide to British, Colonial, Indian, and American Hospitals, Dispensaries, Nursing and Convalescent Institutions. and Asylums. Price 5s. Published by The Scientific Press (Limited), 428, Strand, W.C.

**NOTICE.—The Index for Vol. XII. (ending September 1892) is on sale at the Office of this Journal, price 2½d., post free.**

### Notes and News.

### CONSTRUCTION NOTES.

Small-pox having broken out in the Hemsworth district in Yorkshire, the rural sanitary authority have decided to erect a fever hospital. Mr. Wrightson (formerly of Cusworth Hall, Yorkshire), has generously offered £300 for this purpose. The building will be known as he Wrightson Fever Hospital.

### SCRAPS AND GLEANINGS.

A Pasteur Institute is about to be established at Lisbon.

The Duke of Fife sets a noble example in charity work, as he himself subscribes to 800 charities annually.

A fine of £2 was lately imposed by the Leith magistrates on a lodging house keeper of that locality who had concealed a case of measles in his house.

Folkestone has had an overflow of tramps lately. The workhouse being overfull, it was found necessary to accommodate the arrivals elsewhere.

Over 700 tons weight of butter from Australia arrived last week at the Royal Albert Docks; the value of which commodity is valued at £68,000.

The number of students in the University of Berlin during last term was registered at 4,876, out of which number 1,254 are studying for medicine.

The committee of the Boscombe Infirmary report that the balance owing to the new building fund, which amounted to £255, was paid off last year.

It is suggested to supplement the staff of the West Norfolk and Lynn Hospital with trained nurses, the want of such being felt in the neighbourhood.

Mr. F. H. Walmsley was last month elected medical superintendent of the Metropolitan Asylum for Idiots, situate at Darenth, near Dartford.

The Duke of Westminster contributed £600 during last season to the Chester Infirmary from 1s. tolls for each person who is shown over Eaton Hall.

Amongst the wonders of the Chicago Exhibition of guns the "Krupp" gun will be found, which can throw shot a distance of 16 miles, and is 82 feet long.

Visiting cards of iron are the latest novelty in Germany. These little iron plates are of astonishing thinness, and the name of the visitor is done in silver.

Lord Brassey has become the president of Dr. Barnardo's Homes for the years 1893-4. The 7th June is fixed for the 27th annual meeting of the institution.

In Pennsylvania, Sunday newspapers have been suppressed by the Supreme Legal Tribunal. Republican States, after all, have their liberty restricted sometimes.

The committee of the Colchester Hospital report that the £1,000 anonymous gift received by them for that institution, has been invested in India Three Per Cent. Stock.

A hotel in Hamburg has been built of bricks made of compressed wood; these bricks are said to be as hard as iron, and have the further advantage of being declared fireproof.

The Queen has forwarded £50 towards the National Lifeboat Institution, of which society her Majesty is patron. The Prince of Wales will preside at the next annual meeting.

Large new buildings are to be erected in Berlin for the housing of "The Imperial Health Department," a site for which has already been purchased in the Klopstockstrasse.

Lady Cholmley has presented a further donation of £10 to the Malton Dispensary. Mr. Aspland contributed £17 14s. as the proceeds of an entertainment for the benefit of that institution.

The old-fashioned copper balls for cisterns is to be superceded by a glass globe, which has certain advantages over its predecessors in that it is neither corrodible, nor has it poisonous qualities.

A valuable present of clothing has again been received from Miss Coode, of the Launceston branch of the Cornish Needlework Guild, for distribution among the patients of the Launceston Infirmary.

An American paper points out that antiseptic solutions should be regularly applied to tooth-brushes which are left exposed to the air, as they are thus liable to absorb any disease germs floating about.

A series of concerts and entertainments has been arranged for at the Herbert College, Woolwich, under the direction of Surgeon-Major Gubbins. Over 400 patients were present at the last entertainment.

A new law in Germany orders chemists to make a marked difference in the respective shapes of bottles containing drugs for external or internal use, thus avoiding the danger of misapplication of remedies.

The Russian Black Sea Fleet is to carry out naval manœuvres on a very extensive scale in the spring. Experiments with torpedoes will be tried, amongst which will be tried blowing up old vessels by this means.

The Warneford Hospital Committee reports that as a precautionary measure against their anticipated increase of expenditure, consequent on the enlargement of the hospital, they have invested a legacy of £5,000.

The late Sir Joseph Whitworth devoted some £500,000 to educational and charitable purposes. The Whitworth Institute at Darley, planned by himself, includes reading and recreation rooms, pleasure grounds, swimming baths, and hospital.

At the annual meeting of the friends and subscribers to the Darlington Hospital, the Committee again solicited the help of the subscribers in preventing persons able to pay for medical attendance obtaining gratuitously the benefits of the institution.

The Princess of Wales being the patron of this charity, H.R.H. the Duke of York, H.R.H. the Duchess of Fife, H.R.H. the Princess Christian, H.R.H. the Princess Frederica, H.R.H. the Duchess of Teck, the Lord Mayor, Sir Frederick Leighton, Mr. Henry Irving, and many other members of the nobility have readily accorded their patronage to a concert which will be held in May at St. James's Hall on behalf of the Chelsea Hospital for Women.

At the annual meeting of the National Orthopædic Hospital it was stated that the income for the past year shows a considerable increase on that of the year 1891, a considerable sum having been received for and on behalf of patients. A legacy of £880 has been received towards the building fund, and also a donation of £500; but there is a balance still owing on this account of £1,950. The nursing arrangements under the present matron appear to give great satisfaction.

**For the Book World of Medicine and Science, and Medical Appointments, see pages VIII., IX., X., and XI. overleaf.**

